# Ovarian dose prediction after ovarian transposition in cervical cancer: comparing three models and the impact of geometric distance on predictive performance

**DOI:** 10.3389/fonc.2026.1906826

**Published:** 2026-09-11

**Authors:** Xiaogang An, Meng Jiao, Zhi-Wei Dong, Fei Teng, Li-Na Zhang

**Affiliations:** Department of Radiotherapy, Affiliated Hospital of Hebei University, Baoding, China

**Keywords:** cervical cancer, deep learning, dose prediction, ovarian transposition, random forest

## Abstract

**Purpose:**

To compare three models for predicting mean ovarian dose after ovarian transposition in cervical cancer patients and identify geometric factors affecting prediction reliability.

**Methods:**

We analyzed 100 ovaries from 51 patients using three models: a log-linear model (dose vs. distance), a random forest (RF) incorporating multiple geometric features, and a 3D U-Net for voxel-wise dose prediction. Models were validated using patient-wise 5-fold cross-validation. Accuracy was assessed by mean absolute error (MAE), median absolute error (MedAE), root mean square error, tolerance rates, and Bland-Altman analysis. A sliding-window residual standard deviation curve determined the distance at which prediction uncertainty increased, with subgroup analyses performed accordingly.

**Results:**

RF achieved the lowest MAE (0.803 Gy) and MedAE (0.365 Gy), followed by the log-linear model (MAE: 0.886 Gy) and 3D U-Net (MAE: 0.890 Gy). The log-linear model showed the narrowest limits of agreement, suggesting greater stability. At ±0.5 Gy tolerance, RF significantly outperformed the log-linear model in our cohort (59% vs. 41%, *p* < 0.05). A turning point was identified at 17.1 mm. In the normal-geometry subgroup (distance ≥17 mm, n=71), RF showed the highest accuracy and consistency (MAE: 0.390 Gy, 95% LoA: –0.97 to 0.98 Gy). In the difficult-geometry subgroup (distance <17 mm, n = 29), all models exhibited markedly larger errors (MAE >1.8 Gy). For high-risk classification (dose >6 Gy), RF achieved an AUC of 0.956.

**Conclusion:**

3D U-Net underperformed with limited data, whereas RF, with the lowest MAE, offered a practical alternative; the log-linear model showed the narrowest LoA, suggesting greater stability. All models showed substantially reduced predictive performance below 17 mm, where manual planning should take priority. This threshold may serve as an alert for performance degradation, but external validation is required before clinical use.

## 1 Introduction

Cervical cancer is among the most common malignancies of the female reproductive system. With advances in treatment, the long-term survival of young patients has improved considerably, making preservation of ovarian function an important therapeutic consideration (1). Pelvic radiotherapy is a key component of postoperative adjuvant treatment, but it inevitably exposes the ovaries to radiation, leading to premature ovarian failure, infertility, and endocrine disorders (2). To reduce this damage, ovarian transposition is often performed to relocate the ovaries to a high position outside the pelvic cavity, away from the high-dose target volume (2, 3). Nevertheless, even after transposition, the ovaries may still receive a non-negligible dose. The actual dose depends on several factors, including the distance between the ovaries and the planning target volume (PTV), their spatial relationship, and the beam configuration, optimization objectives, and other factors (4–6).

Even with ovarian transposition, a considerable proportion of patients still have ovarian doses exceeding the functional preservation threshold. For example, Gay et al. (2022) reported that among 31 transposed ovaries, although the majority (64.5%) were located outside the PTV, only 16.1% had a mean dose below 6 Gy, a threshold associated with a moderate risk of ovarian failure (6). Relocating the ovaries outside the PTV alone does not guarantee a safe dose, underscoring the need for accurate pre-plan prediction of ovarian dose to help identify high-risk patients early, guide individualized plan design, and counsel patients about the risk of ovarian damage.

Several geometric parameters have been linked to ovarian dose. Lv et al. (2019) recommended placing the ovary at least 1.12 cm above the iliac crest to achieve lower doses; Gay et al. (2022) found a strong negative correlation between the craniocaudal distance to the sacral promontory or iliac crest and mean ovarian dose; and Yoshihiro et al. (2018) reported correlation coefficients of 0.81–0.86 between the shortest ovary-PTV distance and mean dose in postoperative VMAT (4–6). These findings suggest that distance is a promising predictor. However, the studies mentioned above are descriptive and did not develop generalizable predictive models.

These findings motivate a straightforward approach: building simple, physics-based models. For example, one could test a regression model using only the ovary-PTV distance. Furthermore, incorporating multiple geometric and volumetric features may enable feature-based machine learning models (e.g., random forest, gradient boosting) to improve prediction accuracy. However, none of the above models can predict voxel-level dose distributions. In contrast, many deep learning models have proven to be powerful tools for three-dimensional dose prediction, with 3D U-Net and its variants showing particularly promising results across multiple cancer sites (7–19). Nevertheless, these studies mainly focus on architectural innovations and overall accuracy (20–31); to our knowledge, none have systematically evaluated dose prediction specifically for the transposed ovary. Moreover, deep learning models generally require large training datasets and substantial computational resources, and their lack of interpretability limits clinical acceptance (7).

Ovarian dose prediction also faces several practical challenges. First, the low proportion of patients receiving ovarian transposition leads to a scarcity of high-quality training data. Second, the protection threshold (approximately 5-6 Gy (6, 32, 33)) is only about 10% of a typical pelvic prescription (45–50 Gy) and falls within the same order of magnitude as the error ranges reported in dose prediction studies (from sub-Gy to several Gy) (7, 26, 34–36). Consequently, even a small absolute error may translate into a substantial relative error, increasing the clinical difficulty. Third, protecting the ovaries requires a steep dose fall-off at the target edge, and variations in planner strategies can cause large dose differences for the same geometry. All these factors make model learning more challenging.

In summary, existing 3D dose prediction models are data-hungry, resource-intensive, and poorly interpretable. For low-dimensional prediction tasks (e.g., predicting a single dosimetric parameter), simpler, more interpretable models may offer advantages in small-sample settings, but their predictive accuracy and stability require systematic evaluation.

Given the above, this study used a limited clinical dataset to build and compare three types of models for pre-plan prediction of mean ovarian dose: a simple regression model, a feature-based machine learning model, and a 3D U-Net that provides full dose distributions. We compared their predictive accuracy and stability, focusing on exploring the relationship between prediction error and ovary-PTV distance to identify key geometric factors affecting model performance. The results are expected to guide model selection in small-sample settings and to define the safe application boundaries of these models.

## 2 Materials and methods

### 2.1 Patients and radiotherapy planning

This study was approved by the institutional ethics committee (approval number [HDFYLL-KY-2023-172]). We retrospectively identified 51 patients with cervical cancer who underwent ovarian transposition followed by adjuvant pelvic radiotherapy between January 2015 and December 2020. All structures were contoured by a single experienced radiation oncologist. During ovarian transposition, both ovaries were surgically fixed in a high position (paracolic gutter or intraperitoneal cavity) to displace them away from the high-dose target volume. Forty-nine patients had both ovaries present, and two patients had only one ovary each, resulting in a total of 100 ovaries. All patients received volumetric modulated arc therapy (VMAT) with a prescription dose of 45 Gy in 25 fractions. The clinical planning objective was to keep the maximum ovarian dose as low as possible, ideally ≤ 5 Gy, while ensuring adequate coverage of the clinical target volume (CTV).

### 2.2 Data collection and feature extraction

We exported CT images, structure files (RTSTRUCTURE), and dose files (RTDOSE) from the treatment planning system (TPS). These files were directly used as inputs for the 3D U-Net model. Meanwhile, using in-house Python scripts, we extracted four raw geometric features from the structure files: the shortest distance between the ovary and the planning target volume (PTV), the ovary volume, the shortest distance from the ovary to the body surface, and the distance between the ovary and the PTV centroid.

In addition, the effective overlap index (EOI) was calculated to quantify the spatial overlap between the ovary and the PTV. The EOI was derived from two components:

1. Beam’s-eye view (BEV) component: the 360° treatment arc was discretized into 2° steps (consistent with the angular sampling interval in the TPS). The angles where the ovary projection overlapped the PTV projection were summed and divided by the total arc length.
2. Axial component: the number of CT slices on which the ovary and PTV overlapped along the superior-inferior direction divided by the total number of slices containing the ovary.

The EOI was defined as the product of these two components.

Based on the raw features, we created additional derived features: the inverse, logarithm, and square of the distance; the logarithm and square root of the volume; the distance-to-volume ratio; the relative depth of the ovary; and the depth-volume interaction term. Together with the original features, these formed an initial candidate feature set for subsequent feature selection.

### 2.3 Model development

#### 2.3.1 Simple models

Based on physical intuition, we constructed two single-variable regression models: a log-linear model using the natural logarithm of the ovary-PTV distance ( $D_{mean}=a\cdot ln(d)+b$), where *D_mean_* is the mean ovarian dose and *d* is the ovary-PTV distance and a linear model using the EOI ( $D_{mean}=a\cdot \text{EOI}+b$). Both were fitted using ordinary least squares.

#### 2.3.2 Machine learning models

##### 2.3.2.1 Feature selection

We selected a fixed set of features based on Spearman correlation (|r| > 0.3) and physical interpretability. The four features were EOI, the natural logarithm of the distance, depth-volume interaction, and distance-to-volume ratio. They represent complementary physical dimensions: EOI quantifies the spatial overlap between the ovary and PTV, which affects the difficulty of dose sparing in VMAT; log-transformed distance captures the exponential dose falloff with distance; depth-volume interaction reflects that smaller, more superficial ovaries are easier to spare; and distance-to-volume ratio accounts for the increased scatter risk when larger ovarian volumes are close to the PTV. A nested sensitivity analysis confirmed the robustness of this selection (**Supplementary Table S3**).

##### 2.3.2.2 Model comparison and hyperparameters

Patient-wise five-fold cross-validation (training: validation = 4:1) was used to evaluate nine regression models: EN (Elastic Net), Ridge, BR (Bayesian Ridge), KNN (k-nearest neighbors), GB (Gradient Boosting), XGB (XGBoost), LGB (LightGBM), RF (Random Forest), and a voting ensemble (Ens). For RF, we performed a coarse grid search on the full dataset using mean absolute error (MAE) and the coefficient of determination (R²), then fixed the selected values for all folds (150 trees, maximum depth of 6, minimum samples split of 5, and minimum samples leaf of 3). A nested sensitivity analysis (**Supplementary Table S3**) confirmed that these parameters were near-optimal across folds, with negligible performance difference (*p* = 0.719).

#### 2.3.3 3D U-Net

**Figure 1:**
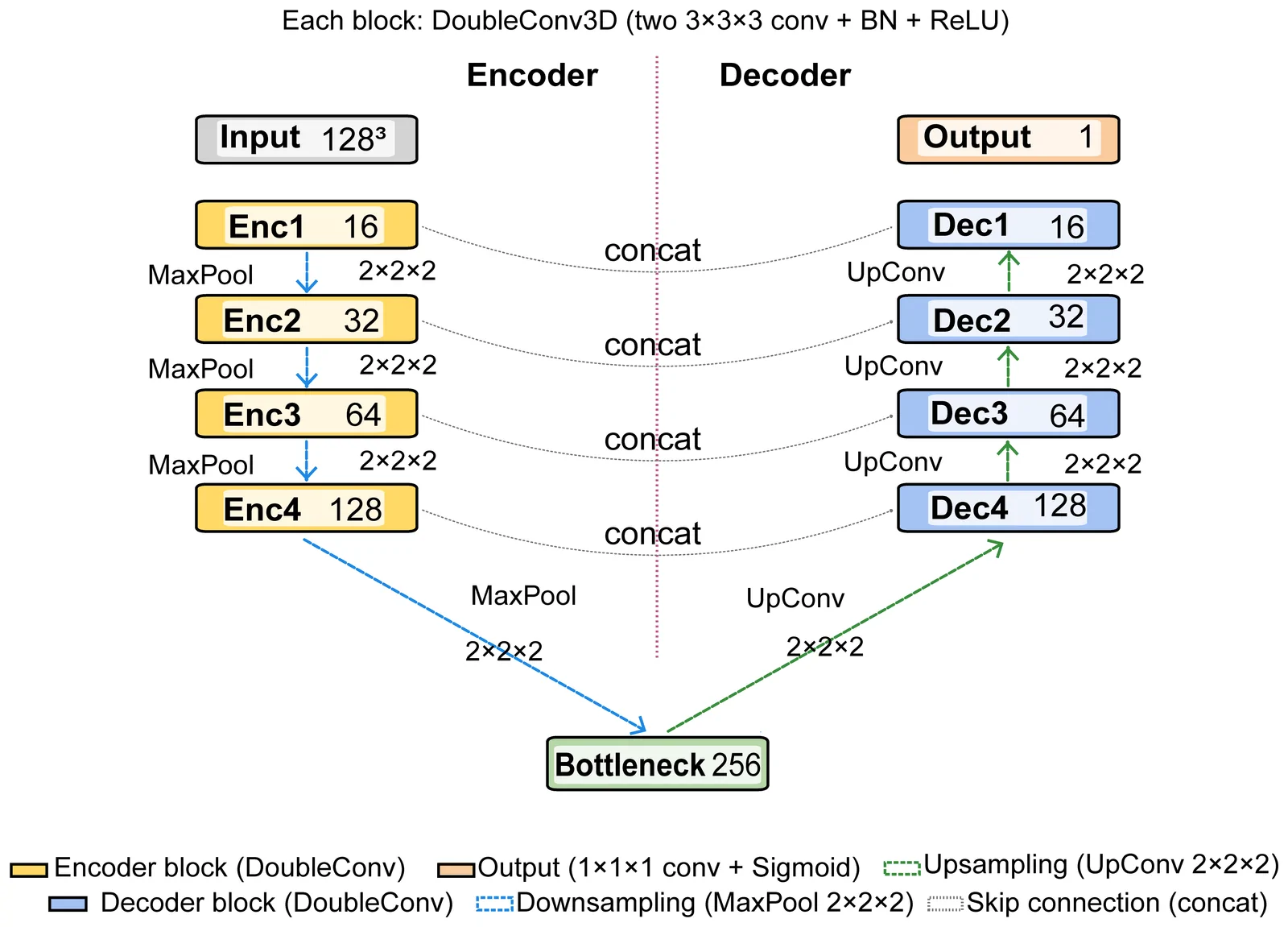
Schematic architecture of the 3D U-Net used for voxel-wise dose prediction.

To explore the feasibility of deep learning under a small-sample scenario, we constructed a 3D U-Net primarily for comparative purposes; it was not extensively tuned. **Figure 1** illustrates the schematic architecture of the model.

##### 2.3.3.1 Input and preprocessing

The input consisted of CT images and 10 regions of interest (ROI) masks (PTV, both ovaries, bladder, rectum, small bowel, colon, spinal cord, left and right femoral heads). Each ROI mask was treated as an independent binary channel. The total number of input channels was 1 (CT) + 10 (ROI) = 11. All data were resampled to an isotropic voxel spacing of 2.5 × 2.5 × 2.5 mm³ and cropped/padded to a fixed size of 80 × 256 × 256 voxels. The output was a single-channel 3D dose distribution with the same resolution as the input.

##### 2.3.3.2 Network architecture

The network followed the standard 3D U-Net design with four down-sampling and four up-sampling levels. The initial number of feature channels was 16. After each down-sampling operation (max-pooling), the channel count doubled, giving 16, 32, 64, and 128 for the four encoder levels. The bottleneck had 256 channels. During up-sampling, the channel count halved after each step (128 → 64 → 32 → 16), and skip connections concatenated the corresponding encoder features. All convolutional layers used 3 × 3 × 3 kernels, followed by batch normalization and ReLU activation.

##### 2.3.3.3 Training strategy

The loss function was voxel-wise mean squared error (MSE). The Adam optimizer was used with a learning rate of 1 × 10^-4^, weight decay of 1 × 10^-5^, and a batch size of 1. Training proceeded for up to 500 epochs, with early stopping (patience 15 epochs) based on the validation loss; the model with the lowest validation loss was saved. Within each cross-validation fold, the training set was randomly split (8:2) into a sub-training and a validation set for early stopping. Online data augmentation was applied: random horizontal flips (left-right and anterior-posterior) with a probability of 0.5, and addition of Gaussian noise (standard deviation 0.02) to the CT images with a probability of 0.2. All augmentations were applied simultaneously to the input CT and the corresponding dose labels. No augmentation was used for the validation set.

##### 2.3.3.4 Post-processing

After prediction, the mean dose was extracted from the predicted 3D dose distribution within the ovary mask and used as the final output for comparison with the simple model and the machine learning model. Training was performed using the same patient-wise five-fold cross-validation (random seed 42) as used for the machine learning models.

### 2.4 Post-hoc geometric subgroup analysis

Using a sliding-window residual standard deviation analysis (**Supplementary Figure S2**) on the pooled residuals, we identified a distance threshold of ≈17 mm. Ovaries were divided into difficult (< 17 mm) and normal (≥ 17 mm) geometry subgroups. Sensitivity analysis with a 10 mm threshold is provided in **Supplementary Table S1**.

### 2.5 Evaluation and statistics

#### 2.5.1 Cross-validation

All models were evaluated using patient-wise five-fold cross-validation (the ovaries of the same patient always stayed in the same fold; training: validation = 4:1). Performance metrics are reported as the mean ± standard deviation across the five folds. These results represent internal estimates from our single-center cohort.

#### 2.5.2 Regression metrics

Mean absolute error (MAE), median absolute error (MedAE), root mean square error (RMSE), Pearson correlation coefficient (r), Bland-Altman 95% limits of agreement (LoA), and the proportions of predictions falling within ±0.5 Gy, ± 1 Gy, and ±2 Gy of the true values (tolerance rates).

#### 2.5.3 Classification metrics

High-risk classification was based on a true dose >6 Gy (a threshold associated with moderate risk of ovarian failure). Accuracy, sensitivity, specificity, positive predictive value (PPV), negative predictive value (NPV), and the area under the receiver operating characteristic curve (AUC) were calculated.

#### 2.5.4 Statistical methods

As the absolute error differences were not normally distributed (Shapiro-Wilk test, *p* < 0.001), non-parametric tests were used. Overall differences among the three models were assessed with the Friedman test. Pairwise comparisons were performed using the Wilcoxon signed-rank test, with *p*-values adjusted for multiple comparisons using the Bonferroni method (significance level α = 0.05/3 ≈ 0.0167). Confidence intervals were estimated via bootstrapping with 2000 resamples.

## 3 Results

### 3.1 Overall prediction performance and statistical comparison

**Figure 2:**
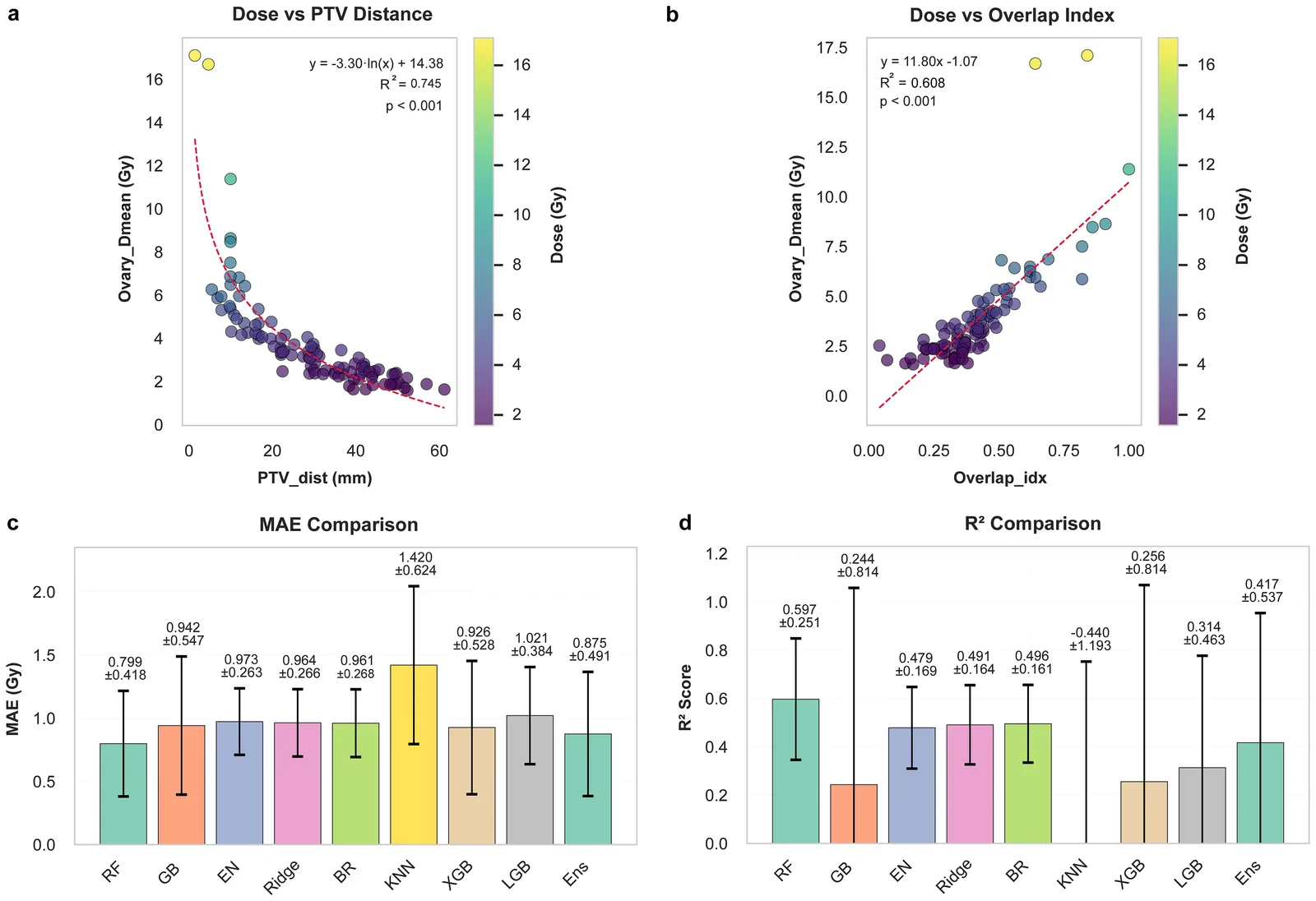
Model pre-selection. **(a, b)** Fitting and comparison of simple models; **(c, d)** Five-fold cross-validation averaged performance of machine learning models, shown as MAE and R².

Among the simple models, the log-linear model using the natural logarithm of the ovary-PTV distance ( $D_{mean}=a\cdot \ln(d)+b$) outperformed the linear model using the EOI (R² = 0.745 vs. 0.608) as shown in **Figures 2a, b**. Moreover, because the distance parameter is easier to obtain in clinical practice, we selected the log-distance model for final evaluation.

For machine learning, we evaluated nine regression models (EN, Ridge, BR, KNN, GB, XGB, LGB, RF, and Ens) using patient-wise five-fold cross-validation. RF achieved the lowest MAE (0.803 Gy) and the highest coefficient of determination (R² = 0.594), so we selected it as the representative model for traditional machine learning (**Figures 2c, d**).

**Figure 3:**
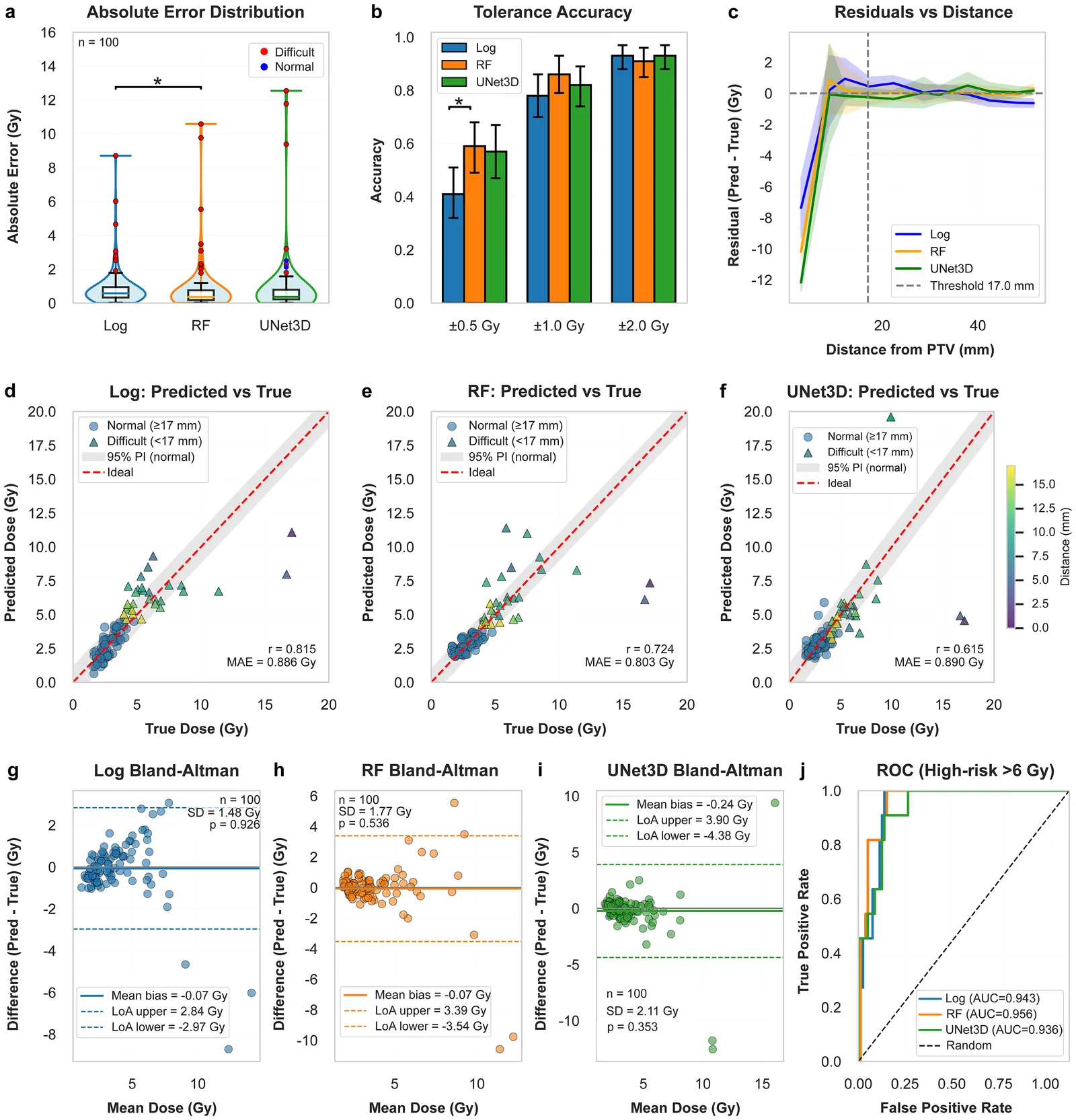
Performance comparison of three models and the impact of distance on model performance. **(a)** Distribution of absolute prediction errors for the three models (red markers indicate geometrically challenging cases); **(b)** Comparison of accuracy under different clinical tolerance thresholds (± 0.5, ± 1, ± 2 Gy); **(c)** Residuals (with 1-SD band) as a function of distance (the dashed vertical line indicates the threshold separating normal from geometrically challenging geometry); **(d–f)** Scatter plots of predicted vs. measured dose, color-coded by distance (darker colors represent smaller distances); **(g–i)** Systematic bias and clinical agreement (Bland-Altman analysis) for the three models; **(j)** Receiver operating characteristic (ROC) analysis for the three models. *p < 0.05. UNet3D denotes the three-dimensional U-Net model.

**Table 1** summarizes the overall performance of the three final models (simple log – Log, RF, and 3D U-Net) on the 100 ovaries. RF gave the lowest MAE (0.803 Gy) and MedAE (0.365 Gy), followed by Log (0.886 Gy and 0.583 Gy) and U-Net (0.890 Gy and 0.371 Gy). Log had the smallest RMSE (1.474 Gy), indicating that its predictions were the most stable, with few large outliers. In contrast, U-Net had the highest RMSE (2.114 Gy), suggesting occasional large errors. The boxplot (**Figure 3a**) shows long tails of absolute errors for all three models; the outliers (red points) mostly came from the difficult-geometry subgroup (distance <17 mm). Log also showed the strongest linear correlation (Pearson r = 0.815), followed by RF (r = 0.724) and U-Net (r = 0.615) as shown in **Figures 3d–f**. Bland-Altman analysis (**Figures 3g–i**) gave mean biases close to zero for all models (Log and RF: –0.07 Gy; U-Net: –0.24 Gy), none of which were statistically significant (Wilcoxon test, *p* > 0.05), indicating no systematic bias.

**Table 1:** Overall predictive performance of the three models and statistical comparisons.

Model	MAE (Gy)	95% CI of MAE	MedAE (Gy)	RMSE (Gy)	*p*-value (vs Log) ^+^	*p-*value (vs UNet) ^+^	Effect size (r)
Log	0.886	[0.685, 1.147]	0.583	**1.474**	–	0.410	–
RF	**0.803**	[0.533, 1.149]	**0.365**	1.760	0.026	0.027	0.262 (vs Log)/0.270 (vs UNet)
3D U-Net	0.890	[0.562, 1.297]	0.371	2.114	0.410	–	0.149 (vs Log)

^+^ Pairwise Wilcoxon signed-rank test with Bonferroni correction. Friedman test overall *p* = 0.030. Bold indicates best performance.

Because the absolute error differences were not normally distributed (Shapiro–Wilk test, *p* < 0.001), we used non-parametric tests. The Friedman test showed a significant overall difference among the three models (χ² = 7.02, *p* = 0.030). Pairwise Wilcoxon signed-rank tests with Bonferroni correction (**Table 1**) revealed that RF had significantly lower MAE than Log (*p*_corr = 0.026, effect size r = 0.262), while no significant differences were found between RF and U-Net or between Log and U-Net (*p*_corr > 0.05).

### 3.2 Clinical tolerance analysis

As shown in **Figure 3b**, at the strict tolerance of ±0.5 Gy, RF achieved a significantly higher accuracy (59%) than Log (41%), with a 95% bootstrap confidence interval for the difference of [0.07, 0.30]. At ±1.0 Gy, RF still had the highest accuracy (86%), but the difference with Log was not statistically significant (95% CI for the difference: [–0.01, 0.17]). At ±2.0 Gy, all three models exceeded 90% accuracy, meaning that most prediction errors were within 2 Gy.

### 3.3 Impact of geometric distance on prediction performance

The residual-versus-distance plot (**Figure 3c**) shows that when the ovary-PTV distance exceeded about 17 mm, residuals approached zero and their spread stabilized. For distances below 10 mm, residuals increased sharply. In the 17–30 mm interval, the Log model exhibited slight overestimation, whereas the U-Net model showed slight underestimation, with a trend reversion beyond 30 mm. In contrast, the RF model fluctuated around zero throughout the entire range above 17 mm.

Rounding the turning point (≈17.1 mm) to 17 mm, we divided the data into a difficult-geometry subgroup (<17 mm, 29 ovaries) and a normal-geometry subgroup (≥17 mm, 71 ovaries). **Table 2** presents the performance of the three models in these subgroups. In the normal-geometry subgroup, RF achieved the lowest MAE (0.390 Gy), significantly lower than Log (0.501 Gy, *p* = 0.013) and U-Net (0.491 Gy), and the narrowest 95% limits of agreement (LoA = [–0.97, 0.98] Gy). Thus, RF gave the highest accuracy and best individual consistency in this subgroup. In the difficult-geometry subgroup, all models had much higher MAEs (1.8–1.9 Gy) and much wider LoAs (± 5–8 Gy), making them unreliable.

**Table 2:** Geometric subgroup performance and agreement based on the 17-mm threshold.

Geometry	Model	N	MAE (Gy)	MedAE (Gy)	RMSE (Gy)	95% LoA (Gy)	Effect size vs Log (r)
Difficult (<17 mm)	Log	29	1.827	1.286	2.579	[-5.17, 5.12]	–
	RF	29	1.813	0.637	3.176	[-6.57, 6.06]	0.151 (*p* = 0.429)
	3D U-Net	29	1.868	0.617	3.779	[-8.24, 6.36]	0.187 (*p* = 0.325)
Normal (≥17 mm)	Log	71	0.501	0.486	0.585	[-1.23, 1.06]	–
	RF	71	**0.390**	**0.322**	**0.492**	**[-0.97, 0.98]**	0.294 (*p* = 0.013)
	3D U-Net	71	0.491	0.359	0.680	[-1.30, 1.38]	0.104 (*p* = 0.381)

In the normal-geometry subgroup, the MAE of RF was significantly lower than that of the log model (*p* = 0.013, Wilcoxon signed-rank test).

The stratified scatter plots (**Figures 3d–f**) further illustrate that points in the normal-geometry subgroup cluster tightly around the ideal line (*y* = *x*), with RF showing the most concentrated distribution and the narrowest 95% prediction interval (**Table 2**). In the difficult-geometry subgroup, points deviate more as the distance decreases, and the color gradient (darker for smaller distances) indicates larger deviations.

A sensitivity analysis using a 10 mm threshold (**Supplementary Table S1**) gave consistent results: for the 14 ovaries with distance <10 mm, all models had MAE >2.5 Gy and LoA > ± 8 Gy; for the normal-geometry subgroup (≥10 mm), RF remained the best (MAE = 0.469 Gy, LoA = [–1.28, 1.34] Gy).

### 3.4 Systematic bias and agreement

Bland-Altman analysis (**Figures 3g–i**) confirmed mean biases near zero for Log and RF (–0.07 Gy) and U-Net (–0.24 Gy), none of which were significant (Wilcoxon test, *p* > 0.05). Log had the narrowest 95% LoA ([–2.97, 2.84] Gy), followed by RF ([–3.54, 3.39] Gy) and U-Net ([–4.38, 3.90] Gy), which agrees with the RMSE results. The subgroup LoAs in **Table 2** further show that RF’s LoA narrowed to ±0.98 Gy in the normal-geometry subgroup, while all models had LoA wider than ±5.1 Gy in the difficult-geometry subgroup.

### 3.5 High-risk patient identification

Although our primary goal was continuous dose prediction, the model outputs can also support clinical risk stratification. Using a true dose >6 Gy as the classifier for high risk, RF achieved the highest AUC in the overall cohort (0.956), followed by Log (0.943) and U-Net (0.936) (**Figure 3j**). RF showed a sensitivity of 81.8% and a specificity of 94.4%; Log had the same sensitivity (81.8%) but a slightly lower specificity (89.9%). In the normal-geometry subgroup (≥17 mm), no patient had a true dose >6 Gy, precluding meaningful high-risk classification. In the difficult-geometry subgroup (<17 mm), AUC values dropped to 0.72–0.78 (RF: 0.783, Log: 0.717, U-Net: 0.753), indicating moderate discriminative ability. **Supplementary Table S2** lists the detailed classification metrics.

### 3.6 Feature importance

**Figure 4:**
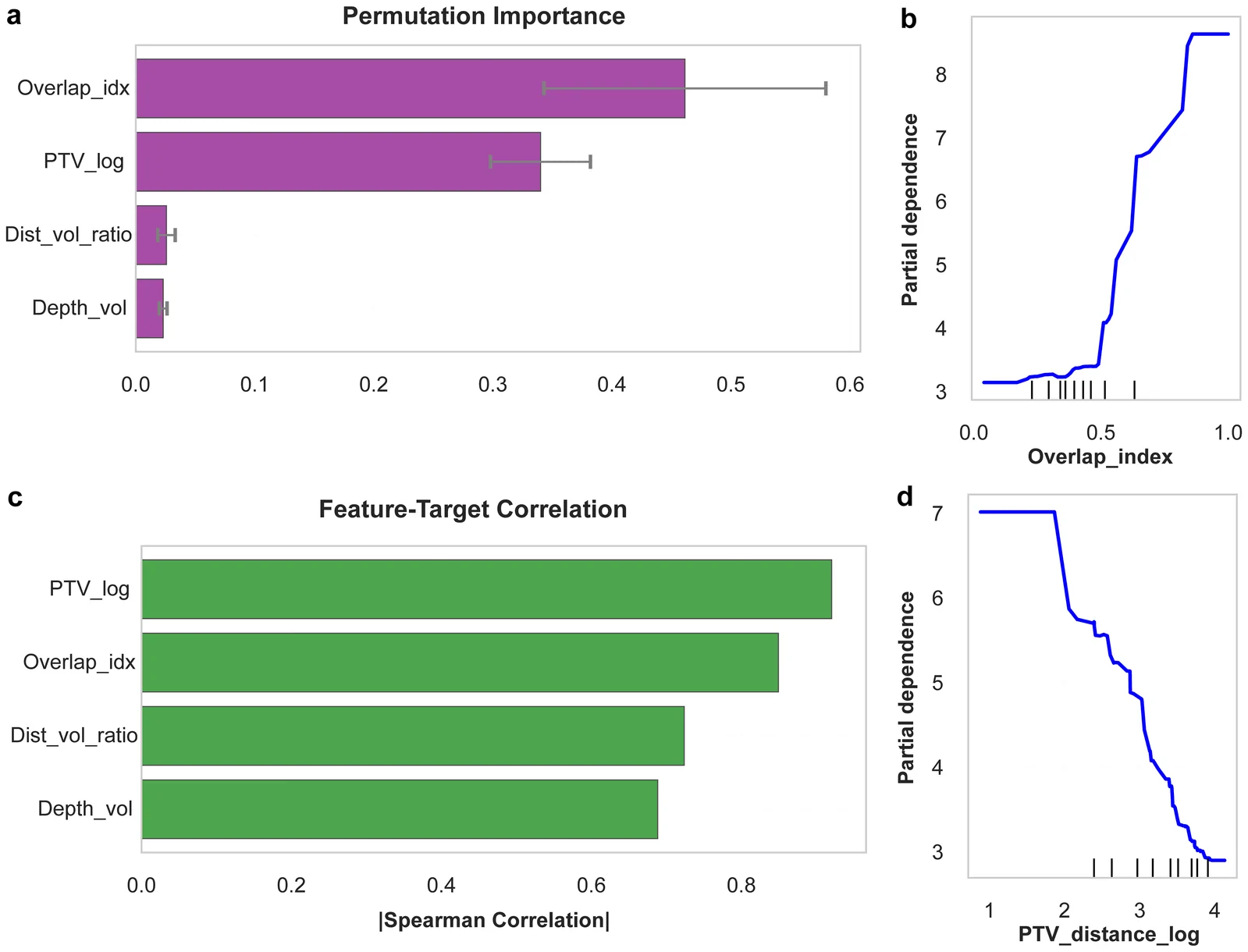
Feature importance analysis based on the random forest (RF) model and the influence of key features on predictions. **(a)** Permutation importance of the four most important features; **(b)** Partial dependence plot for the overlap index; **(c)** Spearman’s rank correlation coefficients between features and the target (ovary mean dose); **(d)** Partial dependence plot for the logarithm of distance.

Feature importance analysis (**Figure 4a**) identified the EOI and the logarithm of distance as the two most important predictors. Partial dependence plots (**Figure 4b**) showed that the predicted dose increased slowly with EOI up to about 0.5, then rose sharply. The dose decreased approximately linearly with the logarithm of distance (**Figure 4d**). The feature-target Spearman correlation ranking (**Figure 4c**) also confirmed that distance and EOI are strongly associated with the mean ovarian dose.

## 4 Discussion

### 4.1 Principal findings

In this study, we compared three methods for predicting mean ovarian dose after ovarian transposition in cervical cancer patients: a distance-based log-linear model (Log), a multi-feature random forest (RF), and a 3D U-Net. In our single-center cohort, RF achieved the lowest MAE. The log-linear model showed the greatest stability, while the 3D U-Net achieved comparable accuracy. Model performance was influenced by ovary-PTV distance, with all three models showing markedly degraded accuracy below 17 mm.

### 4.2 Key geometric predictors

Previous studies have consistently demonstrated a strong association between the ovary-PTV distance and ovarian dose. Yoshihiro et al. (2018) reported negative correlations (R = 0.81–0.86) that varied with planning technique; Gay et al. (2022) found that the distance to the sacral promontory explained 51.6% of the variance in mean dose; and Lv et al. (2019) identified lateral distance as an independent predictor for achieving a maximum dose ≤4 Gy (odds ratio 0.377) (4–6). Consistent with these reports, our RF feature importance analysis confirmed that distance is a key predictor. More importantly, we found that the effective overlap index (EOI) was even more important than distance, likely because the multi-arc nature of VMAT makes the projection overlap angle a major contributor to the integral dose.

Regarding the form of the dose-distance relationship, Yoshihiro et al. (2018) proposed a linear fit ( OMD=−1.11·d+9.8, d in cm) for distances of 20–75 mm (4). Over that far-field range, the curve is relatively flat, so a linear approximation is reasonable. Our data included much shorter distances (down to 1.4 mm) where the dose fall-off is steep. Over the full range of 1.4–61.2 mm, a log-linear function ( $D_{mean}=-3.3\cdot \ln(d)+14.38$) provided a better fit. Dose drops quickly near the target edge and more slowly farther away. The two models are not contradictory but complementary. The linear form works well for ovaries far from the PTV, while the log-linear form is essential for accurate prediction in the near-target region (<20 mm), where prediction errors are largest. This difference does not invalidate the work of Yoshihiro et al. but rather reflects the different distance ranges studied. For clinical decision-making, the most challenging cases are those with ovaries close to the PTV, and there the log-linear model is clearly superior.

RF outperformed the log-linear model in MAE and MedAE, partly because it uses the EOI. In VMAT, the ovary receives dose from multiple beam directions. A larger EOI means the ovary is “seen” by the PTV over a wider angular range, thereby increasing the integral dose. This geometric insight is not captured by distance alone and explains why EOI was the most important feature in RF.

### 4.3 Positioning 3D U-net relative to RF and log

Deep learning models are widely used for dose prediction across multiple cancer sites. A survey of recent literature shows that for organs at risk (OARs), mean dose prediction errors typically range from 0.84 to 2.94 Gy, depending on the OAR size, location, and planning complexity (16, 26, 34–36). For example, in head and neck cancer, Osman and Tamam (2022) reported mean dose errors of approximately 2.44 Gy for parotids, esophagus, and larynx (24), while the Cascade 3D U-Net (Liu et al., 2021) achieved a non-zero dose MAE of 2.50 Gy (26). In prostate cancer, OAR dose errors are generally within 1–2% of the prescription dose (Kajikawa et al., 2019; transfer learning study (19)). For breast cancer, deviations from clinical plans are within 1.0–1.5% (17). In cervical cancer VMAT, a large comparative study (Wu et al., 2024) reported a whole-body MAE of 0.94% of the prescription dose (50 Gy), with large variation across OARs; small bowel MAE was 2.3–2.6%, and femoral head errors were as high as 6–7% (34). Our 3D U-Net achieved a MAE of 0.890 Gy (95% CI: 0.562–1.297) for mean ovarian dose, which is approximately 2.0% of our prescription dose (45 Gy). This value falls well within the typical OAR error range reported in the literature (0.84–2.94 Gy).

Among the three models compared in this study, RF numerically achieved the highest accuracy (MAE = 0.803 Gy), outperforming both the log-linear model and the 3D U-Net (MAE = 0.890 Gy). Unlike the point estimate from RF and log-linear models, the 3D U-Net provides full 3D dose distributions and DVH data, which are essential for plan evaluation. Thus, while the U-Net may not be ideal for point-wise mean dose prediction, its spatial output offers clear clinical value when dose-volume constraints guide planning.

The relatively lower accuracy of the 3D U-Net compared with RF and log-linear models likely stems from two factors. First, deep learning models are inherently data-hungry; with only 51 patients (100 ovaries), the training cohort was too small for the U-Net to realize its full potential, though five-fold cross-validation ensured its performance remained within the literature-reported acceptable range. Second, the U-Net outputs a full 3D dose distribution, yet we only extracted the mean dose within the ovary mask—effectively using a high-dimensional model to predict a low-dimensional quantity, which introduces noise from many irrelevant voxels and further reduces sample efficiency. Whether larger datasets would substantially improve U-Net performance for this specific task warrants further investigation.

To our knowledge, this is the first systematic comparison of these three modeling approaches for ovarian dose prediction in transposed ovaries. In data-scarce clinical settings, RF offers a practical combination of accuracy and interpretability. The 3D U-Net, while less accurate for point-wise mean dose prediction, retains clinical value through its full 3D dose output.

### 4.4 Defining a performance alert threshold

The residual-versus-distance plot (**Figure 3c**) shows that prediction errors increase sharply when the ovary-PTV distance falls below about 17 mm. This turning point was identified using a sliding-window residual standard deviation analysis. In our dataset, it separated cases with better performance (MAE ~0.5 Gy, LoA ~ ± 1 Gy) from those where predictions were substantially less accurate (MAE >1.8 Gy, LoA > ± 5 Gy). This threshold has not been externally validated and should not be used for clinical decision-making. Instead, we propose viewing it as an alert for potential performance degradation: when distance falls below 17 mm, prediction errors tend to increase noticeably, suggesting that manual verification should be considered.

Our data-driven 17 mm threshold agrees with a simple physical estimate. Given a prescription dose of 45 Gy and a target ovarian dose below 5 Gy, assuming a typical dose gradient of about 2 Gy/mm near the PTV edge, a lateral distance of approximately 20 mm would be needed to achieve this dose drop. The similarity between this estimate (≈20 mm) and our threshold (≈17 mm) suggests that the observed turning point is not purely a statistical artifact but may reflect an underlying physical constraint. This aligns with the clinical observation that protecting ovaries within this distance often requires compromising target coverage. Below this distance, it becomes extremely difficult, if not impossible, to keep the ovarian dose below 5 Gy, regardless of planning optimization.

More importantly, such difficult geometry not only poses physical challenges but also amplifies variability in planner decisions. When facing these geometrically challenging cases, different planners may adopt different trade-offs. Some sacrifice target coverage or conformality to lower the ovarian dose, while others accept a higher ovarian dose to ensure target prescription. These trade-offs are likely to vary even more across institutions, where different planning protocols, dose constraints, and optimization preferences further diversify the observed dose–distance relationship. Such strategy differences, combined with the steep dose gradient at the PTV edge, cause large inter-patient variability in the delivered ovarian dose at similar anatomical distances. Consequently, the distance-dose relationship becomes noisy and highly dependent on planning strategy, making it very difficult for any prediction model (simple regression, RF, or 3D U-Net) to learn a stable mapping. This may explain the substantially reduced performance of all three models in the difficult-geometry subgroup (distance <17 mm).

### 4.5 Limitations and future directions

We acknowledge several limitations. This is a single-center, exploratory study with only 51 patients (100 ovaries). All conclusions, particularly the absolute performance metrics and the 17 mm threshold, require independent validation in larger, multi-center cohorts. We lacked a separate external test set. The reported metrics (e.g., RF MAE = 0.803 Gy) come from 5-fold cross-validation without a fully held-out test set, so they may be slightly optimistic. These values should be viewed as internal benchmarks. The 17 mm threshold was derived from our own residual standard deviation curve and may differ across institutions because of variations in contouring, planning, or dosimetry. We therefore provide the full methodological pipeline (sliding-window residual SD analysis) so that other centers can recalculate the threshold on their own data. The 3D U-Net was trained with empirically selected hyperparameters to avoid overfitting and reduce computational cost in this small-sample setting. Whether further tuning or pretraining would improve performance requires investigation with larger datasets. Furthermore, our models predict dose rather than ovarian function directly, as no hormonal or fertility follow-up data were available.

We also explored the models’ performance in a secondary binary classification task (identifying ovaries with a true mean dose >6 Gy). RF achieved an AUC of 0.956 in the overall cohort, indicating good discriminative ability. However, in the difficult-geometry subgroup (<17 mm), the AUC values for all three models dropped to 0.72–0.78. This decrease is likely due to the small sample size, with only 11 high-risk ovaries. Therefore, these classification results should be interpreted with caution and validated in larger, more balanced cohorts.

Based on these findings, we propose several future directions. Multi-center external validation is needed to test the 17 mm threshold and RF model across different treatment planning systems and patient populations. Transfer learning from non-transposed cases (larger datasets of conventional cervical cancer plans with ovaries *in situ*) could provide a useful source domain for pretraining, followed by fine-tuning on transposed ovaries. Incorporating dosimetric features, such as the “PTV-only” dose distribution shown by Ma et al. (2020) (29), may further improve deep learning dose prediction and could be explored for ovarian dose prediction.

## 5 Conclusions

The 3D U-Net was constrained by limited clinical data and performed no better than the log-linear model, whereas RF achieved the lowest MAE numerically and may serve as a practical alternative. All models showed substantially degraded performance when the ovary-PTV distance fell below 17 mm, where automated predictions became unreliable and manual planning was prioritized. This distance threshold may serve as an exploratory indicator of performance degradation, but independent external validation is required before clinical use. Our findings provide evidence-based guidance on model selection in pre-planning ovarian dose prediction for cervical cancer patients under data scarcity.

## Data Availability

The data analyzed in this study is subject to the following licenses/restrictions: Access is restricted due to privacy and ethical reasons; data are available from the corresponding author upon reasonable request. Requests to access these datasets should be directed to anxiaogang@hbu.edu.cn.
