# Supplementary material for: Ovarian dose prediction after ovarian transposition in cervical cancer: comparing three models and the impact of geometric distance on predictive performance
Source: Front Oncol. 2026 Sep 11;16:1906826. doi: 10.3389/fonc.2026.1906826 (PMC13612307; doi:10.3389/fonc.2026.1906826)
Supplement: Supplementary file 1 [file DataSheet1.docx]

Supplementary Material

# 1 Supplementary Figures and Tables

## 1.1 Supplementary Tables

**Supplementary Table S1. Sensitivity analysis: geometric subgroup performance and agreement based on the 10‑mm threshold.**

| Geometry | Model | N | MAE (Gy) | MedAE (Gy) | RMSE (Gy) | 95% LoA (Gy) |
| --- | --- | --- | --- | --- | --- | --- |
| **Difficult** (<10 mm) | Log | 14 | 2.578 | 1.756 | 3.461 | [-7.69, 5.92] |
|  | RF | 14 | 2.853 | 1.506 | 4.405 | [-9.55, 8.13] |
|  | 3D U-Net | 14 | 3.367 | 1.395 | 5.408 | [-12.17, 8.57] |
| **Normal** (≥10 mm) | Log | 86 | 0.610 | 0.534 | 0.759 | [-1.42, 1.56] |
|  | RF | 86 | **0.469** | **0.328** | **0.666** | **[-1.28, 1.34]** |
|  | 3D U-Net | 86 | 0.487 | 0.360 | 0.660 | [-1.29, 1.31] |

Note: This table presents the sensitivity analysis using a 10‑mm cutoff for subgroup definition. Consistent with the primary analysis (17‑mm threshold), RF showed the best performance in the normal‑geometry subgroup, whereas all models exhibited substantially larger prediction errors in the challenging‑geometry subgroup (MAE >2.5 Gy, LoA width >±8 Gy).

**Supplementary Table S2. Performance in identifying high‑risk patients (threshold >6 Gy).**

| Subset | Model | Accuracy | Sensitivity | Specificity | PPV | NPV | AUC | AUC 95% CI |
| --- | --- | --- | --- | --- | --- | --- | --- | --- |
| **Overall (n=100)** | Log | 0.890 | 0.818 | 0.899 | 0.500 | 0.976 | 0.943 | [0.889, 0.982] |
|  | RF | 0.930 | 0.818 | 0.944 | 0.643 | 0.977 | 0.956 | [0.907, 0.989] |
|  | 3D U-Net | 0.940 | 0.455 | 1.000 | 1.000 | 0.937 | 0.936 | [0.871, 0.982] |
| **Difficult (<17 mm, n=29)** | Log | 0.621 | 0.818 | 0.500 | 0.500 | 0.818 | 0.717 | [0.520, 0.899] |
|  | RF | 0.759 | 0.818 | 0.722 | 0.643 | 0.867 | 0.783 | [0.596, 0.946] |
|  | 3D U-Net | 0.793 | 0.455 | 1.000 | 1.000 | 0.750 | 0.753 | [0.534, 0.928] |

**Supplementary Table S3. Nested cross‑validation analysis of feature selection and hyperparameter optimization for the Random Forest model.**

| Fold | Global MAE (Gy) | Nested MAE (Gy) | ΔMAE (Gy) | Selected features | Optimal hyperparameters |
| --- | --- | --- | --- | --- | --- |
| 1 | 1.078 | 0.948 | 0.129 | ptv_min_distance; effective_overlap_index; distance_times_volume; ovary_relative_depth  (consistent across all folds) | n_est=150, depth=6, split=5, leaf=3 |
| 2 | 1.469 | 1.475 | –0.005 |  | n_est=200, depth=6, split=5, leaf=1 |
| 3 | 0.420 | 0.413 | –0.008 |  | n_est=150, depth=6, split=5, leaf=3 |
| 4 | 0.650 | 0.540 | 0.110 |  | n_est=150, depth=8, split=2, leaf=1 |
| 5 | 0.376 | 0.818 | –0.443 |  | n_est=100, depth=8, split=2, leaf=1 |
| Mean ± SD | 0.799 ± 0.467 | 0.839 ± 0.415 | –0.040 ± 0.233 |  | — |

Note: Paired t‑test (Global vs Nested MAE): mean Δ = –0.040 Gy, 95% CI [–0.329, 0.249], *p* = 0.719. Jaccard similarity of selected features across folds = 1.000. The fixed hyperparameters used in the primary analysis (n_estimators = 150 (3/5), max_depth = 6 (3/5), min_samples_split = 5 (3/5), min_samples_leaf = 3 (2/5)) fell within the optimal range identified across folds.

## 1.2 Supplementary Figures

**Supplementary Figure S1.** Representative examples of the 3D U‑Net model (two patients, four ovaries in total). Three conditions are defined: A = geometrically challenging + prediction failed; B = geometrically normal + prediction successful; C = geometrically normal + prediction failed. (a) Geometric conditions for the four ovaries, annotated as A, B, or C (see panel a for ovary‑specific labeling); (b) Dose‑volume histograms (DVHs) for the two patients; (c) Transverse views of the 3D dose distribution and dose difference maps for the two patients; (d) Histograms of dose differences for the two patients.

**Supplementary** **Figure S2**. Determination of thresholds for normal versus challenging geometry. Residual standard deviation is plotted against the ovary‑PTV distance using a sliding‑window analysis. The dashed vertical line indicates the selected distance threshold.
